# The combination of astragalus injection and ambroxol hydrochloride in the adjuvant treatment of COPD: a systematic review and meta-analysis

**DOI:** 10.1038/s41598-023-49421-6

**Published:** 2023-12-12

**Authors:** Zubing Zhou, Lele Yang, Chao Hu, Rui Gao, Xiaobo Zhang, Tao Shen

**Affiliations:** 1 College of Basic Medicine, Chengdu University of Traditional Chinese Medicine, Chengdu, 610075 China; 2 Chengdu University of Traditional Chinese Medicine, Chengdu, 611137 China; 3 Chengdu Integrated TCM and Western Medicine Hospital, Chengdu, 610095 China

**Keywords:** Diseases, Medical research

## Abstract

Chronic obstructive pulmonary disease (COPD) is a severe condition that leads to premature mortality and places a significant financial burden on healthcare systems. An adjunctive therapy in COPD includes the simultaneous administration of astragalus injection and ambroxol hydrochloride. Despite its widespread use, the effectiveness of this combined approach in COPD treatment has not been systematically evaluated. Thus, we conducted a systematic review and meta-analysis to assess the efficacy of combining astragalus injection with ambroxol hydrochloride as an adjuvant treatment for COPD. Six electronic databases were used to search for relevant randomized controlled trials, and data analysis was conducted using Review Manager 5.4. A total of 14 randomized controlled trials were included, involving 1070 patients who met the criteria. The results of the meta-analysis indicated that the combination of astragalus injection with ambroxol hydrochloride as an adjuvant treatment can improve various clinical parameters in patients with COPD compared to conventional treatment alone. These parameters include the clinical effective rate (OR = 5.44, 95% CI 3.51–8.43, I^2^ = 0%), partial pressure of oxygen in artery (MD = 1.12, 95% CI 0.87–1.36, I^2^ = 5%), partial pressure of carbon dioxide in artery (MD = − 1.43, 95% CI − 1.65 to − 1.21, I^2^ = 0%), forced expiratory volume in one second (MD = 0.30, 95% CI 0.18–0.42, I^2^ = 0%), percentage of forced expiratory volume in one second (MD = 16.18, 95% CI 12.60–19.76, I^2^ = 82%), forced vital capacity (MD = 0.33, 95% CI 0.21–0.45, I^2^ = 36%), hemoglobin (MD = − 16.17, 95% CI − 20.84 to − 11.51, I^2^ = 29%), and the ratio of forced expiratory volume in one second to forced vital capacity (MD = 2.51, 95% CI − 0.05 to 5.06, I^2^ = 0%). The combination of astragalus injection and ambroxol hydrochloride could be a selection of COPD patients as an adjuvant treatment. However, further validation is required to evaluate the effectiveness of combining astragalus injection and ambroxol hydrochloride as an adjunctive treatment for patients with COPD.

## Introduction

Chronic obstructive pulmonary disease (COPD) is a global disease with 3.97 million new cases reported in China in 2019. The incidence of COPD tends to increase with age^1^. COPD is a preventable and treatable disease, but airflow restriction is progressive and affects patients' quality of life^1^. At present, the primary treatment methods for COPD include anti-infection medications, cough suppressants, expectorants, bronchodilators, and spasmolytics. Although medical technology continues to improve, COPD is still a significant medical burden^1^.

Ambroxol hydrochloride is a commonly used drug in the adjunctive treatment of COPD^2^. It can effectively alleviate the clinical symptoms and improve lung function in patients with COPD^2^. Astragalus injection is a traditional Chinese medicine injection that can be used in conjunction with conventional treatment to aid in the treatment of COPD^3,4^. The combination of traditional Chinese medicine preparation and conventional treatment can reduce the number of acute exacerbations of COPD and improve the clinical efficacy of COPD treatment^5,6^. Clinical researchers in China have used astragalus injection in combination with ambroxol hydrochloride as an adjuvant treatment for COPD^7^. However, the effects of combining astragalus injection with ambroxol hydrochloride on patients with (COPD) have never been systematically evaluated. To assess the therapeutic efficacy of astragalus injection combined with ambroxol hydrochloride as an adjuvant treatment for COPD, we conducted a systematic review and meta-analysis by summarizing relevant randomized controlled trials (RCTs).

## Methods

This study followed the guidelines of the Preferred Reporting Items for Systematic Reviews and Meta-Analyses (PRISMA) project, and all study procedures were conducted in accordance with PRISMA requirements^8^. The systematic review has been registered on the International Prospective Register of Systematic Reviews (PROSPERO), and the ID is CRD42023421010.

### Information sources and search strategies

The databases used were PubMed, China Biomedical Literature Database (CBM), Wan Fang Database, Embase, Web of Science, and the China National Knowledge Infrastructure (CNKI). The literature search strategy is ("Astragalus injection") AND ("Ambroxol hydrochloride") AND ("COPD" OR "Chronic obstructive pulmonary diseases"). To ensure that no important literature is missed, we conducted a thorough literature search using the strategy shown in Supplementary Material. The retrieval date was set from the database's establishment to October 26, 2023. There were no language restrictions.

### Inclusion and exclusion criteria

Inclusion criteria: (a) Type of study (S): The included study was a randomized controlled trial; (b) Type of participant (P): Adult patients clinically diagnosed with COPD and hospitalized for COPD; (c) Type of intervention (I): The experimental group was given Astragalus injection and Ambroxol hydrochloride and conventional treatment, and the administration was via an intravenous drip; (d) Type of comparator (C): The control group received conventional treatment; (e) Type of prognostic measurement (O): These studies included one of the following outcomes: clinical effective ratio (CER), lung function, and arterial blood gas analysis, hemoglobin.

Exclusion criteria: (a) Unable to extract sufficient data for statistical aggregation; (b) Outcome measurement and conclusion were inconsistent; (c) Duplicate published or duplicated data; (d) The original data was not available.

### The type of the outcome measurement

The clinically effective ratio (CER) was the primary outcome, and forced expiratory volume in one second (FEV1), percentage of forced expiratory volume in one second (FEV1%), forced vital capacity (FVC), partial pressure of oxygen in artery (PaO_2_), partial pressure of carbon dioxide in artery (PaCO_2_), the ratio of forced expiratory volume in one second to forced vital capacity (FEV1/FVC), hemoglobin, were the secondary outcomes.

### Selection of studies and extraction of data

Two reviewers (CH and ZBZ) reviewed potentially eligible studies and documented the selection process independently, and then completed the PRISMA flowchart. The data was independently extracted by (ZBZ and XBZ) using the extraction table. The information retrieved was as follows: Name of the first author, year of publication, study design, the sample size of the trial, the mean age of the participants, the interventions of the experimental group and the control group, duration, and outcomes. Differences in the retrieved information were resolved through consulting with LLY and RG.

### Risk of bias assessment

The quality of the methods in each trial was scored independently scored by two evaluators (LLY and ZBZ), which included seven domains: appropriate sequence generation, hidden allocation, blinding of participants and people, incomplete outcome data, selective reporting, and other biases. Three levels were used to assess the quality of the method: "low bias risk" ( +), "high bias risk" (−), and "uncertain bias risk" (?). We discussed the differences with other researchers (CH, XBZ, and RG) to reach a consensus.

### Data synthesis and analysis

The Review Manager 5.4 was used for the meta-analysis. Dichotomous data were expressed as odds ratio (OR). Continuous data were expressed as mean difference (MD) with a 95% confidence interval when the units of measurement for the outcome variable are the same. When I^2^ > 50% and P < 0.05, there was significant heterogeneity between studies, and a random effects model was used for meta-analysis. When there was no heterogeneity between studies, the fixed effects model was used to summarize OR and MD. Qualitative analysis of data that cannot be included in the meta-analysis.

### Sensitivity analysis

Sensitivity analysis was conducted to assess the robustness of the pooled OR values and the pooled MD values by excluding each trial.

### Risk of publication bias

As more than 10 studies were included, we would test for potential publication bias using a funnel plot.

### Quality assessment of the evidence

Two reviewers (CH and ZBZ) used GRADE prolifer to assessed the grade of each evidence independently. A third reviewer (LLY) determined the differences in the assessments. Four levels of "high", "medium", "low", and "very low" were used to evaluate the quality of evidence. If the sample size of the trial was less than 400, or the pooled values with high sensitivity, the quality of the study would be reduced by one grade.

## Results

### Description of studies

**Figure 1:**
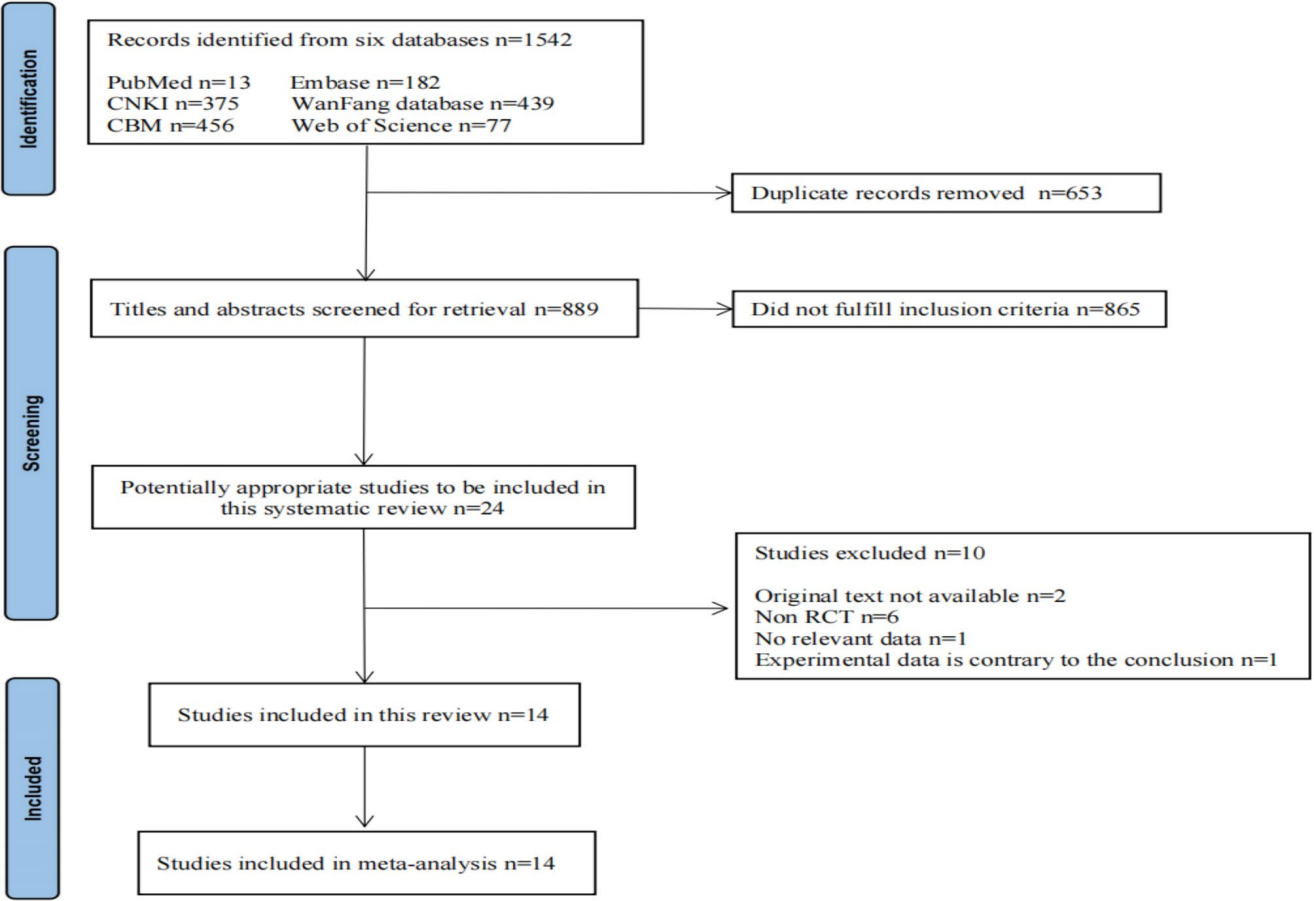
Flow chart of the literature screening process.

We identified 1542 related studies from the databases. Thereafter, we obtained 24 articles after excluding 653 duplicated articles and 865 articles that did not meet the inclusion criteria. After further review of the full text, 14 studies were excluded and 10 randomized controlled trials (n = 1070) were finally included, all of which were from China. The literature selection process is displayed in Fig. 1, and the general characteristics of studies are listed in Table 1.

**Table 1:** Characteristics of the included studies.

Author/year	Country	Study design	Sample size		Mean age		Interventions	Comparator	Dosage		Duration	Outcomes
			T	C	T	C			T	C		
Ibaditi 2017^9^	China	RCT	35	35	61.25 ± 2.18	61.69 ± 2.17	A + B + Bronchodilators, oxygen inhalation, and antibiotics	Bronchodilators, oxygen inhalation, and antibiotics	A:10-20 ml/qd	U	14d	CER; PF; HGB
									B:30 ml/bid			
									Routine			
Dai 2014^3^	China	RCT	29	29	64.28 ± 6.26	64.29 ± 5.96	A + B + Relieving cough, anti-infection, relieving asthma, spasmolysis, expectorant	Relieving cough, anti-infection, relieving asthma, spasmolysis, expectorant	A:20 ml/qd	U	14 d	CER; PF; ABG
									B:30 ml/bid			
									Routine			
Guan 2018^10^	China	RCT	40	40	64.24 ± 6.25	64.25 ± 6.23	A + B + Oxygen and expectorant, relieving asthma, spasmolysis, bronchodilators, antibiotics	Oxygen and expectorant, relieving asthma, spasmolysis, bronchodilators, antibiotics	A:20 ml/qd	U	14 d	CER; PF
									B:30 ml/bid			
									Routine			
Hu 2012^11^	China	RCT	35	35	64.8 ± 8.4	67.4 ± 6.8	A + B + Oxygen and expectorant, relieving asthma, spasmolysis, bronchodilators, antibiotics	Oxygen and expectorant, relieving asthma, spasmolysis, bronchodilators, antibiotics	A:60 ml/qd	U	14 d	CER; PF
									B:30 ml/bid			
									Routine			
Jing 2008^12^	China	RCT	38	36	U	U	A + B + Antibiotics, bronchodilators, oxygen inhalation, and symptomatic supportive treatment	Antibiotics, bronchodilators, oxygen inhalation, and symptomatic supportive treatment	A:60 ml/qd	U	14 d	CER; PF; ABG; HGB
									B:30 ml/qd			
									Routine			
Ni 2013^4^	China	RCT	30	30	65.15 ± 7.92	66.31 ± 8.01	A + B + Oxygen inhalation, relieving cough, anti-infection, relieving asthma, spasmolysis, expectorant	Oxygen inhalation, relieving cough, anti-infection, relieving asthma, spasmolysis, expectorant	A:60 ml/qd	U	14 d	CER; PF
									B:30 ml/bid			
									Routine			
Xie 2012^13^	China	RCT	60	60	73.5 ± 5.8	72.1 ± 6.3	A + B + Spasmolysis, relieving asthma, anti-infection and oxygen therapy	Spasmolysis, relieving asthma, anti-infection and oxygen therapy	A:60 ml/qd	U	14 d	CER; PF; ABG; HGB
									B:30 ml/bid			
									Routine			
Song 2013^14^	China	RCT	38	38	44 ~ 78		A + B + Relieving cough, anti-infection, expectorant, oxygen inhalation and relieving asthma	Relieving cough, anti-infection, expectorant, oxygen inhalation and relieving asthma	A:60 ml/qd	U	14 d	CER; PF
									B:30 ml/bid			
									Routine			
Yu 2013^15^	China	RCT	49	49	48 ~ 77		A + B + Bronchial dilator (terbutaline), glucocorticoids (budesonide)	Bronchial dilator (terbutaline), glucocorticoids (budesonide)	A:60 ml/qd	U	14 d	CER; PF; ABG
									B:30 ml/bid			
									Routine			
Tang 2016^7^	China	RCT	30	30	62.18 ± 3.24	62.56 ± 3.89	A + B + Relieving cough, anti-infection, expectorant, oxygen inhalation, relieving asthma	Relieving cough, anti-infection, expectorant, oxygen inhalation, relieving asthma	A:60 ml/bid	U	14 d	CER
									B:30 ml/bid			
									Routine			
Wang 2020^16^	China	RCT	36	36	61.8 ± 1.2	61.9 ± 1.1	A + B + Bronchodilators, relieving cough and expectorant, and anti-infection	Bronchodilators, relieving cough and expectorant, and anti-infection	A:20 ml/qd	U	14 d	CER; PF; ABG
									B:30 ml/bid			
									Routine			
Wang 2016^17^	China	RCT	43	43	63.6 ± 5.8	63.8 ± 5.9	A + B + Relieving asthma, relieving cough, expectorant, anti-infection, spasmolysis	Relieving asthma, relieving cough, expectorant, anti-infection, spasmolysis	A:60 ml/qd	U	14 d	CER
									B:30 ml/bid			
									Routine			
Zhang 2015^18^	China	RCT	32	32	62.5 ± 8.3	61.8 ± 8.7	A + B + Anti-infection, relieving cough, expectorant, relieving asthma, bronchodilator, spasmolysis	Anti-infection, relieving cough, expectorant, relieving asthma, bronchodilator, spasmolysis	A:60 ml/qd	U	14 d	CER; PF; ABG
									B:30 ml/bid			
									Routine			
Zhang 2017^19^	China	RCT	41	41	62.35 ± 4.27	62.35 ± 4.27	A + B + Oxygen inhalation, bronchodilator, anti-infection, expectorant	Oxygen inhalation, bronchodilator, anti-infection, expectorant	A:60 ml/bid	U	14 d	CER; PF
									B:30 ml/bid			
									Routine			

*U* Unknown, *A* Astragalus injection, *B* Ambroxol Hydrochloride, *CER* Clinical effective rate, *PF* pulmonary function, *ABG* arterial blood gas, *HGB* Hemoglobin.

### Risk of bias in the included studies

**Figure 2:**
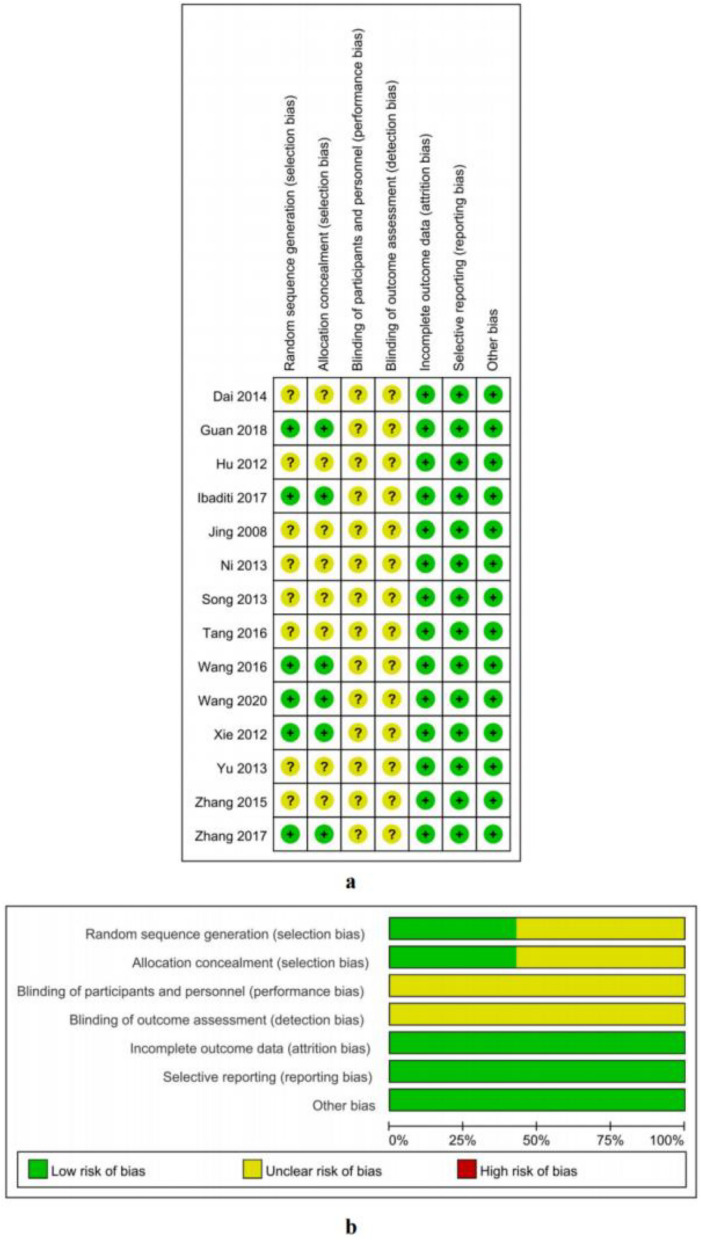
Risk of bias summary and Risk of bias graph.

Figure 2a and Fig. 2b summarized the bias risk assessment for 14 RCTs. From the 14 RCTs, eight studies had no specific methods^3,4,7,11,12,14,15,18^, three studies used random number tables^9,10,13^, one study used random blind selection^16^, one study used a double-blind method^19^, and one study used a lottery for random allocation^17^. None of the 14 studies mentioned blinding the participants, personnel, or assessment outcomes. Data from the 14 studies were complete and did not exist other risks of bias.

### Primary outcome

#### Clinical effective ratio (CER)

**Figure 3:**
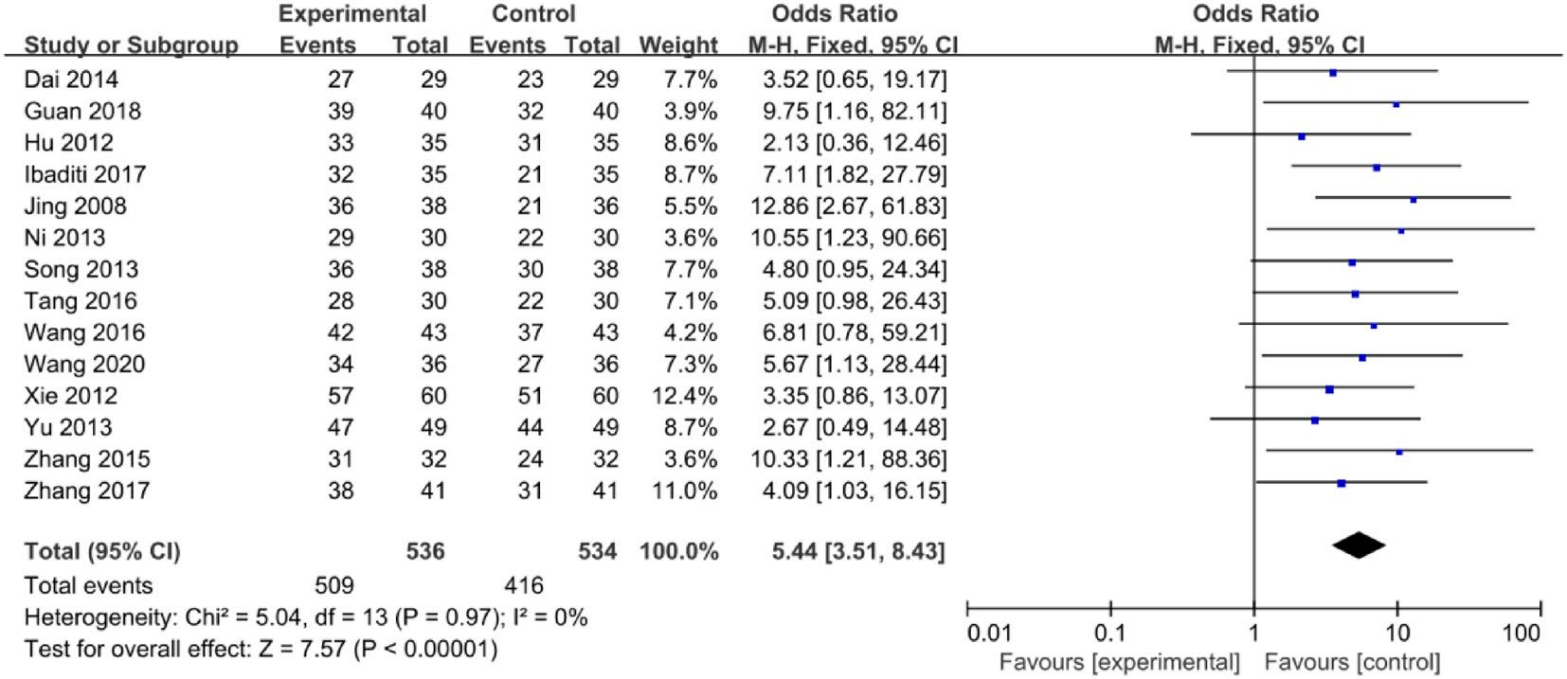
The forest plot of CER.

Fourteen RCTs (N = 1070) reported the effect of astragalus injection combined with ambroxol hydrochloride on the clinical effective ratio (CER) of COPD. CER = (number of obvious effective cases + number of effective cases) / total number of people in each group × 100%. The results of the data analysis show that the combination of astragalus injection and ambroxol hydrochloride injection with conventional treatment significantly improved CER, compared to conventional treatment alone (OR = 5.44, 95% CI: 3.51–8.43, P < 0.00001). There was no heterogeneity among the fourteen studies (P = 0.97, I^2^ = 0%), and a fixed-effects model was used for the meta-analysis (Fig. 3).

### Secondary outcomes

#### Pulmonary function: FVC, FEV1, FEV1/FVC, and FEV1%

**Figure 4:**
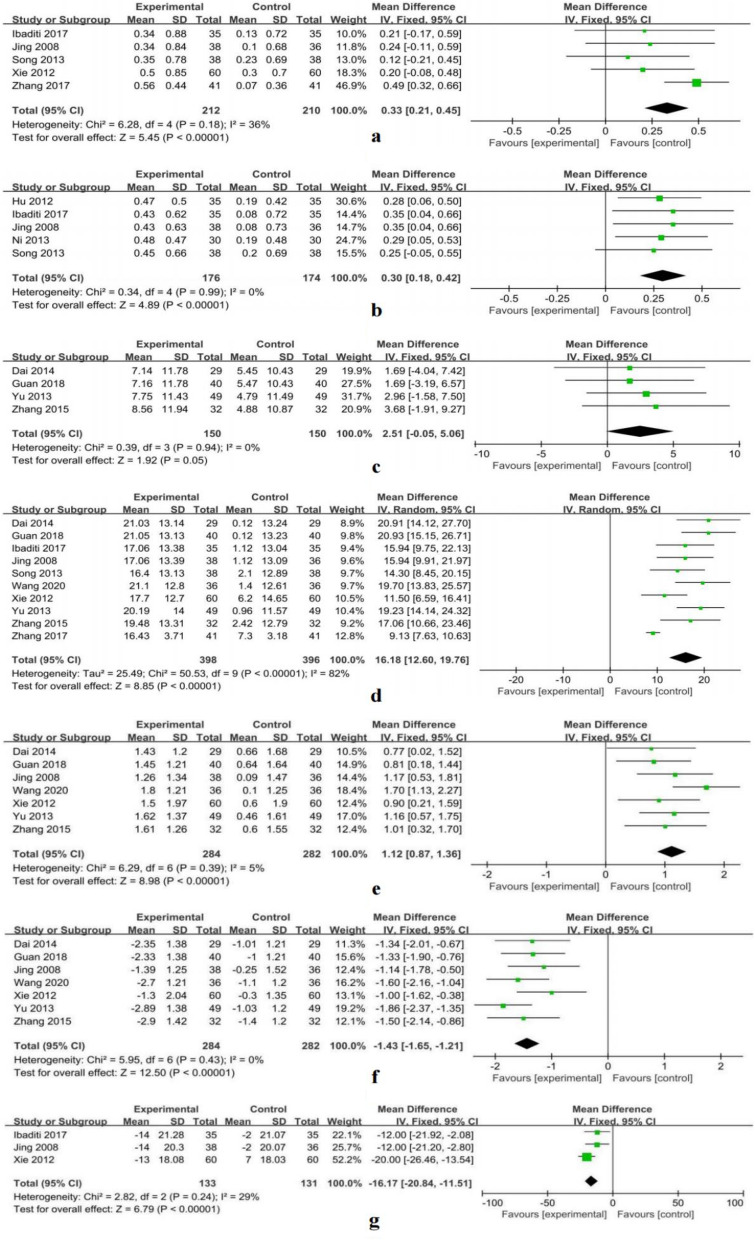
The forest plots of FVC, FEV1, FEV1/FVC, FEV1%, PaO_2_, PaCO_2_, and hemoglobin.

Five RCTs (N = 422) reported FVC^9,12–14,19^. Data analysis revealed that the combination of astragalus injection and ambroxol hydrochloride with conventional treatment can improve FVC in patients with COPD compared to conventional treatment alone (MD = 0.33, 95% CI 0.21–0.45, P < 0.00001). There was no heterogeneity among the five studies (P = 0.18, I^2^ = 36%), and a fixed-effects model was used for the meta-analysis (Fig. 4a).

Five RCTs (N = 350) reported FEV1^4,9,11,12,14^. Data analysis revealed that the combination of astragalus injection and ambroxol hydrochloride as an adjuvant treatment significantly improved FEV1 compared to conventional treatment alone (MD = 0.30, 95% CI 0.18–0.42, P < 0.0001). There was no heterogeneity among the five studies (P = 0.99, I^2^ = 0%), and a fixed-effects model was used for data analysis (Fig. 4b).

Four RCTs (N = 300) reported the FEV1/FVC^3,10,15,18^. Data analysis revealed that the combination of astragalus injection and ambroxol hydrochloride can improve the FEV1/FVC compared to conventional treatment alone (MD = 2.51, 95% CI − 0.05 to 5.06, P = 0.05). No heterogeneity was found among the four studies (P = 0.94, I^2^ = 0%), and data were analyzed using a fixed-effects model (Fig. 4c).

Ten RCTs (N = 794) reported FEV1%^3,9,10,12–16,18,19^. Data analysis revealed that the combination of astragalus injection and ambroxol hydrochloride with conventional treatment significantly improved patients’ FEV1% compared to conventional treatment alone (MD = 16.18, 95% CI 12.60–19.76, P < 0.00001). There was significant heterogeneity among the ten studies (P < 0.00001, I^2^ = 82%), and a random-effects model was used for the meta-analysis (Fig. 4d). Sensitivity analysis revealed that heterogeneity originated from the Zhang 2017 (Table 2), the source of heterogeneity may be the dosage of astragalus injection.

**Table 2:** Sensitivity analysis of FEV1% and FEV1/FVC.

	Pooled values after the each study was omitted			
The omitted study	MD	95% CI	Heterogeneity	Test for overall effect (P)
FEV1%				
Dai 2014	15.7	12.05 ~ 19.35	P < 0.00001, I^2^ = 82%	P < 0.00001
Guan 2018	15.63	12.03 ~ 19.23	P < 0.00001, I^2^ = 81%	P < 0.00001
Ibaditi 2017	16.23	12.35 ~ 20.1	P < 0.00001, I^2^ = 84%	P < 0.00001
Jing 2008	16.23	12.34 ~ 20.11	P < 0.00001, I^2^ = 84%	P < 0.00001
Song 2013	16.41	12.48 ~ 20.35	P < 0.00001, I^2^ = 84%	P < 0.00001
Wang 2020	15.79	12.09 ~ 19.49	P < 0.00001, I^2^ = 82%	P < 0.00001
Xie 2012	16.77	12.73 ~ 20.81	P < 0.00001, I^2^ = 84%	P < 0.00001
Yu 2013	15.81	12.1 ~ 19.52	P < 0.00001, I^2^ = 81%	P < 0.00001
Zhang 2015	16.10	12.27 ~ 19.94	P < 0.00001, I^2^ = 83%	P < 0.00001
Zhang 2017	17.09	14.87 ~ 19.31	P = 0.23, I^2^ = 24%	P < 0.00001
FEV1/FVC				
Dai 2014	2.71	− 0.14 ~ 5.57	P = 0.86, I^2^ = 0%	0.06
Guan 2018	2.82	− 0.18 ~ 5.82	P = 0.89, I^2^ = 0%	0.07
Yu 2013	2.3	− 0.79 ~ 5.39	P = 0.84, I^2^ = 0%	0.15
Zhang 2015	2.2	− 0.67 ~ 5.07	P = 0.91, I^2^ = 0%	0.13

#### Arterial blood gas analysis: PaO_2_, PaCO_2_

PaO_2_ was reported in seven RCTs (N = 566)^3,10,12,13,15,16,18^. Data analysis revealed that the combination of treatments significantly improved patients' PaO_2_ compared to conventional treatment alone (MD = 1.12, 95% CI 0.87–1.36, P < 0.00001). There was no significant heterogeneity among the seven studies (P = 0.39, I^2^ = 5%), and a fixed-effects model was used (Fig. 4e).

PaCO_2_ was reported in seven RCTs (N = 566)^3,10,12,13,15,16,18^. Data analysis revealed that the combination significantly improved patients' PaCO_2_ compared to conventional therapy (MD =  − 1.43, 95% CI − 1.65 to − 1.21, P < 0.00001). There was no heterogeneity among the seven studies (P = 0.43, I^2^ = 0%), and a fixed-effects meta-analysis was used (Fig. 4f).

#### Hemoglobin

Three RCTs (N = 264) reported the effects of astragalus injection combined with ambroxol hydrochloride on the hemoglobin levels of patients with COPD^9,12,13^. Data analysis showed that, compared to conventional treatment alone, the combination therapy significantly down regulated the hemoglobin levels of patients with COPD (MD =  − 16.17, 95% CI − 20.84 to − 11.51, P < 0.00001). There was no heterogeneity among the seven studies (P = 0.24, I^2^ = 29%), and a fixed-effects meta-analysis was used (Fig. 4g).

### Sensitivity analysis

We use sensitivity analysis to assess the robustness of merged data and investigate the sources of heterogeneity. Through sensitivity analysis, it was found that, in addition to FEV1/FVC, the pooled mean difference (MD) values of FVC, FEV1, PaO_2_, PaCO_2_, and the pooled odds ratio (OR) values of CER are robust. Sensitivity analysis results of FEV1% and FEV1/FVC are shown in Table 2.

### Publication bias analysis

**Figure 5:**
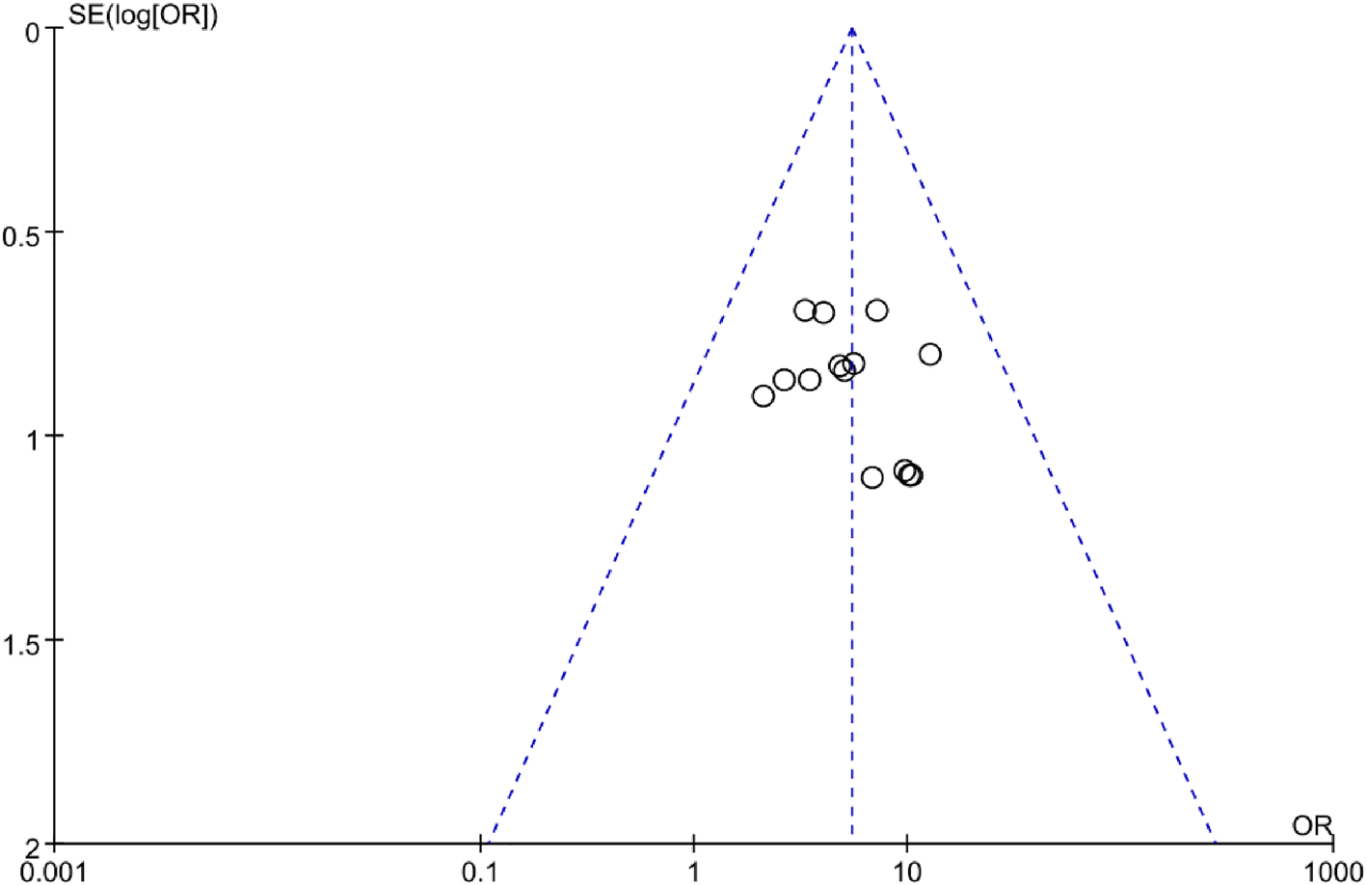
Funnel plot of CER.

As shown in Fig. 5, the funnel plot of the CER is approximately symmetric, indicating no publication bias.

### Quality of evidence

We evaluated the quality of evidence. Due to unclear bias risk in research methods and the medication and dosage of routine therapy are unclear, the quality of evidence for CER, PaO_2_, PaCO_2_ and FVC has been downgraded by two grade. The quality of evidence for FEV1 has been downgraded by two levels due to unclear bias risk in research methods and a sample size of less than 400. Because of the unclear bias risk in research methods, a sample size of less than 400, and the high sensitivity of the pooled MD values, the quality of evidence for hemoglobin, FEV1/FVC, and FEV1% is considered to be very low. The comprehensive results are presented in Table 3, providing a clear depiction of the quality of the evaluated evidence.

**Table 3:** Quality of evidence.

Quality assessment							No of patients		Effect		Quality
No of studies	Design	Risk of bias	Inconsistency	Indirectness	Imprecision	Other considerations	Test	Control	Relative (95% CI)	Absolute	
CER	RCT	Serious^A^	NS	NS	Serious^E^	N	509/536 (95%)	416/534 (77.9%)	OR 5.44 (3.51 to 8.43)	171 more per 1000 (from 146 to 188 more)	⊕⊕OO
14 studies											Low
FEV1/FVC	RCT	Serious^A^	NS	NS	Serious^B, E^	Serious^C^	150	150	–	MD 2.51 higher (-0.05 lower to 5.06 higher)	⊕OOO
4 studies											Very low
FEV1%	RCT	Serious^A^	NS	NS	Serious^D, E^	N	398	396	–	MD 16.18 higher (12.6 to 19.76 higher)	⊕OOO
10 studies											Very low
PaO_2_	RCT	Serious^A^	NS	NS	Serious^E^	N	284	282	–	MD 1.12 higher (0.87 to 1.36 higher)	⊕⊕OO
7 studies											Low
PaCO_2_	RCT	Serious^A^	NS	NS	Serious^E^	N	284	282	–	MD 1.43 lower (1.65 to 1.21 lower)	⊕⊕OO
7 studies											Low
FEV1	RCT	Serious^A^	NS	NS	Serious^B^	N	176	174	–	MD 0.3 higher (0.18 to 0.42 higher)	⊕⊕OO
5 studies											Low
FVC	RCT	Serious^A^	NS	NS	Serious^E^	N	212	210	–	MD 0.33 higher (0.21 to 0.45 higher)	⊕⊕OO
5 studies											Low
Hemoglobin	RCT	Serious^A^	NS	NS	Serious^B, E^	N	133	131		MD 16.17 lower (20.84 to 11.51 lower)	⊕OOO
3 studies											Very low

*NS* Not serious, *N* none.

^A^The methodology of most studies has an unclear bias.

^B^The sample size was less than 400.

^C^The sensitivity analysis revealed a highly sensitive profile.

^D^Heterogeneity test I^2^ > 75% (82%).

^E^The medication and dosage of routine therapy are unclear.

## Discussion

In the study, we conducted a systematic evaluation of the clinical efficacy of astragalus injection combined with ambroxol hydrochloride as an adjuvant treatment for COPD. Additionally, we evaluated the therapeutic impact of this combination on pulmonary function and arterial blood gas analysis.

In terms of clinical efficacy, the combination of astragalus injection with ambroxol hydrochloride as adjuvant treatment significantly improved the CER in COPD. Seven studies obtained CER from clinical symptoms such as cough, phlegm, and shortness of breath, as well as from chest X-rays^4,10,11,14,16,17,19^. Three studies obtained CER based on cough, sputum volume, and pulmonary rales^7,15,18^. Two studies obtained CER according to the Guidelines for Clinical Research on expectorant cough suppressants^12,13^. One study obtained the CER based on improvements in body temperature and coughing^9^. One study did not describe the method of obtaining CER^3^. In future studies, the method of obtaining CER should be standardized, such as the COPD Assessment Test (CAT). In terms of lung function, the combination of astragalus injection and ambroxol hydrochloride can improve FVC, FEV1/FVC, FEV1, and FEV1% in COPD patients as an adjuvant treatment. However, the quality of evidence for FEV1% and FEV1/FVC is very low. In terms of arterial blood gas analysis, the combination of astragalus injection and ambroxol hydrochloride significantly improved the levels of PaO_2_ and PaCO_2_ in patients with COPD.

Although no adverse effects were reported in the 14 studies, early studies indicated that astragalus injection may have some side effects^20^. Hence, further research is needed to determine the safety of combining astragalus injection with ambroxol hydrochloride in COPD patients.

The pathogenesis of COPD is related to chronic inflammation, oxidative stress, and immune imbalance. Cytokine release and oxidative stress are induced by airborne particles 2.5 (PM2.5), which can exacerbate COPD conditions^21,22^. The Th17/Treg ratio in the lungs of COPD patients is unbalanced, and the release of the cytokine IL17 by Th17 cells leads to the exacerbation of COPD symptoms^23^. Astragaloside IV, one of the main components of Astragalus injection, has anti-inflammatory, antioxidant, and immune regulatory effects^24^. Additionally, astragaloside can activate the AMPK/mTOR signaling pathway and reduce lung inflammation and injury induced by PM2.5^25^. Astragaloside IV also inhibits the cellular response of Th17^26^. In clinical studies, ambroxol hydrochloride has been shown to be an effective adjunctive therapy in reducing inflammation, alleviating clinical symptoms, and improving pulmonary function^2^. Traditional Chinese medicine believes that deficiency in the spleen and lungs is one of the causes of COPD^27^. Astragalus can tonify the qi of the lungs and spleen. Clinical studies have shown that astragalus injection can enhance lung function in patients with COPD^28^.These findings might explain the enhanced clinical effectiveness of combining astragalus injection with ambroxol hydrochloride in COPD.

However, this study had some limitations. First, the number of relevant studies included was only 14, all of which were published in China, and did not describe whether these patients were in a stable or acute phase of COPD. Additionally, the overall quality of these studies was not high, which diminished the credibility of the findings. Second, although all the studies used random assignment, only six studies provided specific explanations, which may increase the risk of selection bias. Thirdly, None of the studies indicated whether double-blind trials were conducted, and the overall quality of the 14 included studies was low. Fourth, the pooled mean MD value for FEV1/FVC are highly sensitive, which reduces the quality of the evidence. Furthermore, 14 of the included studies did not specify the use of conventional treatment drugs and dosages, which undermines the credibility of the evidence. In future studies, more clinical trials are needed to verify the clinical efficacy of combining astragalus injection with ambroxol hydrochloride as adjuvant therapy in patients with COPD.

## Conclusion

In conclusion, as an adjuvant treatment, the combination of astragalus injection and ambroxol hydrochloride significantly improves the clinical symptoms of patients with COPD. This suggests that combining astragalus injection with ambroxol hydrochloride could be a viable adjuvant treatment option for patients with COPD. However, due to the limitations of the trials, further validation is required to evaluate the effectiveness of combining astragalus injection with ambroxol hydrochloride for patients with COPD. Additionally, future trials should preferably employ a clear randomization method and incorporate a double-blind test.

### Supplementary Information

Supplementary Information.

## Data Availability

Raw data relevant to the conclusions of this study will be provided by the corresponding authors upon reasonable request.

## Abbreviations

COPD: Chronic obstructive pulmonary disease
FEV1: Forced expiratory volume in one second
FEV1%: Percentage of forced expiratory volume in one second
FVC: Forced vital capacity
FEV1/FVC: The ratio of forced expiratory volume in one second to forced vital capacity
PaO_2_: Partial pressure of oxygen in artery
PaCO_2_: Partial pressure of carbon dioxide in artery
