# Supplementary material for: The combination of astragalus injection and ambroxol hydrochloride in the adjuvant treatment of COPD: a systematic review and meta-analysis
Source: Sci Rep. 2023 Dec 12;13:22077. doi: 10.1038/s41598-023-49421-6 (PMC10716149; doi:10.1038/s41598-023-49421-6)
Supplement: Supplementary file 1 — Supplementary Information. [file 41598_2023_49421_MOESM1_ESM.docx]

Supplementary material 1:

Search strategy in PubMed

＃1 Astragalus plant

＃2 Astragalus propinquus

＃3 Astragalus membranaceus

＃4 Huang qi

＃5 Huang Qi

＃6 Astragalus injection

＃7 (＃1 OR ＃2 OR ＃3 OR ＃4 OR ＃5 OR ＃6)

＃8 Pulmonary disease, Chronic Obstructive

＃9 Chronic Obstructive Lung Disease

＃10 Chronic Obstructive Pulmonary Disease

＃11 COAD

＃12 COPD

＃13 Chronic Obstructive Airway Disease

＃14 Bronchitis

＃15 Chronic Bronchitis

＃16 Chronic Airflow Obstruction

＃17 Pulmonary Emphysema

＃18 (＃8 OR ＃9 OR ＃10 OR ＃11 OR ＃12 OR ＃13 OR ＃14 OR ＃15 OR ＃16 OR ＃17)

＃19 (＃7 AND ＃18)

((((((Astragalus plant) OR (Astragalus propinquus)) OR (Astragalus membranaceus)) OR (Huang qi)) OR (Huang Qi)) OR (Astragalus injection)) AND ((((((((((Pulmonary disease, Chronic Obstructive) OR (Chronic Obstructive Lung Disease)) OR (Chronic Obstructive Pulmonary Disease)) OR (COAD)) OR (COPD)) OR (Chronic Obstructive Airway Disease)) OR (Bronchitis)) OR (Chronic Bronchitis)) OR (Chronic Airflow Obstruction)) OR (Pulmonary Emphysema))

Search strategy in CNKI

(Subject: Huang Qi (precise)) OR (theme: Huang Qi injection (precise)) AND (theme: ambroxol hydrochloride (precise)) AND ((theme: chronic obstructive pulmonary disease (precise)) OR (theme: COPD (precise)) OR (theme: chronic bronchitis (precise)) OR (theme: emphysema (precise)) OR (theme: COPD (precise))
